# Ellagic Acid and Pomegranate (*Punica granatum*) Peel Powder Enhances Growth and Health Performance in Nile Tilapia (*Oreochromis niloticus*)

**DOI:** 10.1155/anu/4057455

**Published:** 2025-12-01

**Authors:** Majid Khanzadeh, Ahmad Farhadi, Andrew G. Jeffs

**Affiliations:** ^1^Department of Animal Biological Product Research Group, Academic Center for Education, Culture and Research (ACECR), Tehran, Iran; ^2^Department of Natural Resources and Environmental Engineering, Shiraz University, Shiraz, Iran; ^3^School of Biological Sciences, The University of Auckland, Auckland, New Zealand

**Keywords:** ellagic acid, gene expression, immune-antioxidant, *O. niloticus*, pomegranate peel powder

## Abstract

Ellagic acid (EA), in pure form or extracted from pomegranate (*Punica granatum*) peel (PP), is a bioactive polyphenol that provides strong anti-inflammatory and antioxidant effects in cultured fish. To investigate the benefit and any adverse effects of PP as a source of EA, and pure EA by assessing growth parameters, digestive enzymes, antioxidant, and immune factors when supplied to 450 juvenile *Oreochromis niloticus* (10.4 ± 0.9 g) over 60 days. Feeding treatments used a standard feed with EA included at three concentrations (i.e., 0.1, 0.15, and 0.2 g kg^−1^) and PP at three concentrations (i.e., 10, 15, and 20 g kg^−1^) and in combination with similar doses (i.e., EP 1 = 50 mg + 5 g kg^−1^, EP 1.5 = 75 mg + 7.5 g kg^−1^, and EP 2 = 100 mg + 10 g kg^−1^ to keep the EA content around 0.1, 0.15, 0.2 g kg^−1^, respectively) as well as a control without inclusion. The final weight (FW) of the fish was significantly increased in most treatments compared to the control group (*p* < 0.05). White blood cell (WBC) counts increased in higher dose treatments for both EA and EP (i.e., EA 0.2 g, EP 1.5, and EP 2). Antioxidant and key digestive enzyme (protease, lipase, and *α*-amylase) activities were generally elevated, with most treatments showing significant (*p* < 0.05) increases in the transcription of glutathione genes or activity of superoxide dismutase (SOD), catalase (CAT), and a decrease in the malondialdehyde (MDA). Immune responses showed significant increases in immunoglobulin M (IgM), lysozyme, and respiratory burst activity (RBA), and the expression of immune genes in several treatments. Notably, EA at 0.2 g and EP 2 elicited a stronger response than the other dosages for these parameters in Nile tilapia. These findings suggest that dietary supplementation with EA, PP, or their combination enhances growth performance, immune responses, and antioxidant capacity in *O. niloticus*, with the EA 0.2 g and EP 2 treatments showing the most pronounced effects.

## 1. Introduction

Maintaining the health of animals in intensive aquaculture rearing systems can represent a significant challenge, with the potential to hinder production through mortality or reduced growth rate. Various solutions are currently used to address these challenges, such as the use of antibiotics for disease management. However, the use of antibiotics has led to several issues, including the development of resistance to pathogens, as well as environmental risks [1]. Furthermore, antibiotics can facilitate the transfer of drug resistance to humans, which has led to widespread legal prohibitions and restrictions on their use [1].

Natural botanical compounds are increasingly being used for promoting the performance of cultured aquatic species, often through their inclusion in feeds, to promote growth, enhance health, boost immunity, and improve disease resistance [2–4]. Among the various medicinal plants for use in humans, pomegranate (*Punica granatum*) is a flavorful medicinal fruit known for its strong antioxidant properties, primarily utilized for juice production [5]. Pomegranate peel (PP), which is a byproduct of the pomegranate industry, accounts for roughly 50% of the entire fruit [6]. The PP has potential as a nutritional supplement in animal feed because it contains high levels of antioxidant compounds, including polyphenols [7] and polysaccharides [8], which have antioxidative, antimicrobial [9], antiatherogenic [10], hepatoprotective [11], and antimutagenic [6] properties. Ellagic acid (EA) is a naturally occurring polyphenol found in a range of fruits, including pomegranates [12], raspberries [13], walnuts [14], and almonds [15]. EA is a derivative of chromene-dione with the formula C_14_H_6_O_8_, which can be found in several forms, including free, glycosylated variants, and as part of ellagitannins [16, 17]. The antioxidant activity of EA is several times greater than that of vitamin E succinate and melatonin [16], being capable of boosting antioxidant enzyme activity, inhibiting lipid peroxidation, and lowering the production of reactive oxygen species (ROS) [17]. Therefore, the use of herbal extracts such as ferulic acid [18], trans-cinnamic acid [19], and p-coumaric acid [20] in animal feed is recognized as a highly effective approach [21].

Nile tilapia, *Oreochromis niloticus*, is extensively cultured globally because of its rapid growth, adaptability to commercial diets, and resistance to environmental stressors [22]. The commercial aquaculture of this species and its adaptability to experimental conditions make it a suitable laboratory species for evaluating the effectiveness of natural compounds for improving health under culture conditions [23].

The positive effect of PP on growth has been reported in farmed animals [24]. Both EA and PP powder have been found to improve growth, antioxidant activity, and immune and antioxidant system genes expression parameters in animals [25, 26]. For example, the inclusion of EA in feeds for yellow catfish, *Pelteobagrus fulvidraco*, has a positive impact on growth, antioxidant capacity, and the expression of genes associated with autophagy (such as atg3, becn1, atg4a, lc3a, atg7, and sqstm1), apoptosis (including smad 2, tgf-*β*, caspase 9, caspase 3, and baxa), and inflammation (interleukins) [27]. Likewise, feeding with PP powder significantly enhanced antioxidant activities in the liver of Nile tilapia [28]. Additionally, the dietary supply of PP decreased the adverse effects of silver nanoparticles (AgNPs), leading to reduced liver and kidney damage and oxidative stress [29]. However, some concerns regarding the potential adverse effects of antinutrients and compounds exist in plant material, including PP, which needs further investigation [30]. Natural compounds, including herbal extracts, can influence the health and well-being of animals at various levels, ranging from gene expression and biochemical processes to growth and survival. Sensitive indicators, such as hematological parameters, are suitable for assessing physiological effects, side effects, and determining the optimal and safe dosage in medicinal plant applications. Therefore, in this study, the effects of PP powder along with EA, and their combination were examined on a wide range of growth parameters, digestive enzymes, antioxidant system, immune-antioxidant activity, and the expression of genes related to immune-antioxidant functions in *O. niloticus* to better understand any potential adverse effect from PP compared to purified EA. We further aimed to investigate whether the immune and antioxidant effects of these feed additives, especially at higher doses, result in adverse effects on growth and the digestive system of the tilapia.

## 2. Materials and Methods

### 2.1. Preparation of Diets and Experimental Design

Ten dietary treatments for feeding Nile tilapia were designed to provide comparable EA doses between pure EA and PP added to feeds (Table 1). Three concentrations of EA (i.e., 0.1, 0.15, and 0.2 g kg^−1^) (based on [25, 31]) and three of PP (i.e.,10, 15, and 20 g kg^−1^) in diet [32, 33] were selected for feed inclusion alongside a control diet without any supplementation. Also, three combined treatments (EP) were formulated to match the EA dose using EA + PP ratios (i.e., EP 1 = 0.05 g + 5 g kg^−1^, EP 1.5 = 0.075 g + 7.5 g kg^−1^, and EP 2 = 0.1 g + 10 g kg^−1^). The formulation for EP was based on the assumption that PP contains ~1% EA [34], and in each treatment, supplying 50% of the total EA from pure EA and 50% from PP powder. The basal feed mixture contained all ingredients (Table 1) except EA or PP. EA powder (Sigma–Aldrich, MO, E2250–1 G) and PP powder (Giyahkala Refinery, Iran) were acquired. The ingredients were mixed and ground using a meat grinder and then extruded into moist 3 mm pellets. The prepared pellets were air-dried, sieved, and kept at 4°C until use. Proximate analyses were undertaken on diets to confirm the extent of the main nutrient content following the addition of the experimental inclusions. Four hundred and fifty juvenile *O. niloticus*, of an average weight of 10.4 ± 0.9 g, were acquired from a commercial fish farm in Semnan, Iran, and transferred into 1000 L fiberglass tanks at the research center. After a 14-day acclimation period, the fish were randomly assigned to 30 tanks (i.e., 15 fish per 150 L fiberglass tank), with three replicate tanks assigned to the 10 feed treatments. The fish were fed twice daily with an amount equivalent to 3% of their total biomass over a 60-day period, with the feeding quantity adjusted every 10 days based on measured changes in the biomass in each tank. The tanks were checked visually 30 min after the feed broadcast to ensure there was no food waste. Fecal matter was removed from each tank by siphoning daily. Additionally, 30% of the water volume in each tank was replaced with fresh water daily. Throughout the study, the fish were kept under a light cycle of 12 h of light followed by 12 h of darkness. The water temperature was maintained at 32 ± 1°C. Optimal environmental conditions were maintained for the fish as confirmed with daily measurements of dissolved oxygen (7.28 ± 0.21 mg L^−1^) and pH (7.46 ± 0.12) using a portable device (AZ86031, AZ instrument, Taiwan), equipped with separate probes for each parameter. Water quality and replacement, temperature, and feeding regimen across all tanks were maintained identically.

### 2.2. Growth Indicators

The fish weights were recorded at the beginning and at the end of the 60-day trial. The growth performance of *O. niloticus* was evaluated by measuring the following parameters [35]:

  $$\begin{array}{c} \text{Weight gain }\left(\text{\% }\right)\;=\frac{\text{Final weight }\left(g\right)-\text{initial weight }\left(g\right)}{\text{Initial weight}}\;\times 100, \end{array}$$

  $$\begin{array}{c} \text{Specific growth rate }\left(\%\text{ day}\right)\;=\frac{\left[Ln\;\left(\text{final weight}\right)-\;Ln\;\left(\text{initial weight}\right)\right]}{\text{Days}}\times \;100, \end{array}$$

  $$\begin{array}{c} \text{Food conversion ratio }\left(g/g\right)=\frac{\text{Feed consumed }}{\text{Weight gain}}, \end{array}$$

  $$\begin{array}{c} \text{Survival rate }\left(\%\right)\;=\frac{\text{Number of survived individuals}}{\text{Initial number of individuals}}\times \;100. \end{array}$$

### 2.3. Blood and Tissue Sampling

At the end of the experiment on day 60, fish were starved for 24 h, and then three fish were randomly chosen from each replicate tank and anesthetized with clove powder at a concentration of 150 mg L^−1^, and ~2 mL blood sample was collected from the caudal vein of each fish. The blood was then divided into two fractions: one was treated with heparin for the evaluation of whole blood parameters, while the other was left nonheparinized to obtain serum. The nonheparinized blood was refrigerated for 12 h to facilitate clot formation. Once clotted, the samples were centrifuged at 3000 × *g* for 15 min at 4°C to isolate the serum, which was subsequently stored at −80°C for future analysis of biochemical, immunological, and antioxidant parameters. To determine the effect of the supplements on parameters, liver tissue and digestive tract from each sampled fish were taken and pooled across tanks (replicates), flash-frozen in liquid nitrogen, and kept at −80°C for later analysis. In order to measure gene expression after blood collection to reduce pain in fish, we used a high dose of clove powder for euthanization (400 mg L^−1^).

### 2.4. Hematological Parameters

Hematological parameters were evaluated using the methodology outlined by Grant [36]. Briefly, the quantification of white blood cells (WBCs) and red blood cells (RBCs) was performed after diluting the samples with Dacie solution, utilizing a Neubauer slide for counting. Hematocrit (HCT) levels were determined through a centrifugation technique, while hemoglobin (HB) concentrations were measured using the cyanomethaemoglobin method [36].

### 2.5. The Serum Biochemical and Antioxidant Parameters

The total protein (TP), globulin (Glb), albumin (Alb), aspartate aminotransferase (AST), alkaline phosphatase (ALP), and alanine aminotransferase (ALT) in serum were evaluated using commercial kits (Pars Azmun, Iran), in accordance with the manufacturer's guidelines. Glucose levels were assessed using the methodologies outlined by Trinder [37].

The measurement of serum malondialdehyde (MDA) was conducted following the protocol established by Yagi [38]. A mixture consisting of 50 µL of serum, 50 µL of normal saline, 100 µL of trichloroacetic acid (TCA), and 25 µL of thiobarbituric acid (TBA) reagent was added to a tube and incubated in a water bath at 95°C for 1 h. The TBA reagent was prepared by dissolving 200 mg of TBA in 30 mL of distilled water and 30 mL of acetic acid. Subsequently, 300 µL of n-butanol was added to each tube, and the samples were centrifuged at 3000 rpm for 10 min. The butanol layer, which separated during centrifugation, was collected and analyzed using a spectrophotometer set at a wavelength of 535 nm, against a reagent blank.

The assessment of serum superoxide dismutase (SOD) and catalase (CAT) was performed utilizing commercial kits (Zellbio, Veltinerweg, Germany), in accordance with a previously published study [39]. The determination of serum SOD measures the conversion of superoxide anions into hydrogen peroxide, while for CAT, the evaluation was based on the rate at which hydrogen peroxide is decomposed. The quantitative colorimetric analysis of glutathione dehydrogenase (GSH) was conducted following the method described by Beutler et al. [40].

### 2.6. Immunological Parameters

The immunoglobulin M (IgM) concentration in serum was measured using an ELISA method, following the protocol established by Cuesta et al. [41]. In summary, 96-well plates were coated with 100 µL of diluted serum samples (1:200) and incubated overnight at 4°C. After blocking with 5% skim milk for 2 h at room temperature, mouse anti-Nile tilapia IgM polyclonal antibody (1:2000) was added and incubated at 37°C for 37 h. Following a secondary incubation with horseradish peroxidase-conjugated rabbit anti-mouse antibody, the reaction was developed using O-phenylenediamine dihydrochloride, and absorbance was measured at 490 nm after terminating the reaction with 25 µL of 2 M H_2_SO_4_.

Serum lysozyme activity was determined using a turbidimetric method with *Micrococcus luteus* as described by Demers and Bayne [42]. Various dilutions of egg white lysozyme were prepared, ranging from 0 to 20 *μ*L mL^−1^ in a 0.1 M phosphate-citrate buffer at pH 8.5 to serve as standards. These standards, along with 25 *μ*L of undiluted serum samples, were added to a 96-well plate in triplicate, followed by the addition of 175 *μ*L of *M. luteus* suspension (75 mg mL^−1^ in the same buffer). The turbidity changes were recorded every 30 s for a total of 5 min at a wavelength of 450 nm using a microplate reader.

To evaluate respiratory burst activity (RBA), 100 µL of heparinized blood was combined with 100 µL of a 0.2% nitro blue tetrazolium (NBT) chloride solution and incubated for 30 min at 25°C. Following this incubation, 100 µL of the blood–NBT mixture was added to 1 mL of dimethylformamide (HCON(CH_3_)_2_). The samples were then centrifuged, and the absorbance of the supernatant was measured at a wavelength of 540 nm [43].

### 2.7. Digestive Enzymes

An aliquot of 50 mM Tris–HCl buffer at pH 7.5 was prepared at a 1:5 weight/volume ratio and added to the digestive tract. The mixture was then homogenized for 1.5 min, after which it was centrifuged at 10,000 × *g* for 20 min at 4°C [44]. To determine protease activity, 20 *μ*L of the supernatant was mixed with 0.5 mL of 2% azocasein in a 50 mM Tris–HCl buffer at pH 7.5 and incubated for 10 min. Following this incubation, 0.5 mL of TCA was added, and the samples were then centrifuged at 6500 × *g* for 5 min, in accordance with the method described by Garcia-Carreno and Haard [45]. The activities of lipase and amylase were assessed following the methodologies previously established by Ahmadifar et al. [46] and Zamani et al. [47].

### 2.8. Gene Expression

Total RNA was extracted from liver tissue using a commercial kit (Sinaclon, Iran) following the manufacturer's protocol. To assess RNA quantity, absorbance was measured at 260 and 280 nm using a nanodrop (Thermo Fisher Scientific, MA), while RNA quality was evaluated through electrophoresis on a 1% agarose gel. For cDNA synthesis, 1 *μ*g of RNA from each sample was converted using the HyperScript First-strand synthesis kit (GeneAll, Korea) and a set of six primers for the associated target genes, as well as *β*-actin, served as the housekeeping gene for normalization purposes (Table 2). The reaction mixture consisted of 1 µL of cDNA, 0.5 µM of each forward and reverse primer, and 5 µL of 2x SinnaSYBR Green HSqPCR mix (Sinaclon, Iran), with the total volume brought to 10 µL by adding molecular-grade water and quantified using StepOne plus Real-Time PCR (Thermo Fisher Scientific, MA). The relative mRNA expression of the specified genes was analyzed using the 2^−*ΔΔ*Ct^ method alongside a standard curve approach as outlined by Livak and Schmittgen [50].

### 2.9. Statistical Analyses

Data analyses were conducted using GraphPad PRISM software (version 10.4.1.627). The normality of the data was checked with Shapiro–Wilk test, and the homogeneity of variance was examined through Levene's test. Following this, one-way ANOVA was performed alongside Tukey's post hoc test to evaluate differences among all the treatments. The results are presented as mean values accompanied by their respective SD. Since the values for the measured parameters among the samples from replicate (tanks) were not significant based on a preliminary nested ANOVA, results were pooled across the tanks as replicates for each treatment. The gene expression data are presented as relative expression, and the control group is considered baseline with relative expression equal to 1.

## 3. Results

### 3.1. Growth Parameters

Fish in all treatments consumed the entire feed during each feeding time, with no difference in feed intake observed among treatments. The administration of PP or its combination with EA at various concentrations did not show any adverse effect on growth parameters in *O. niloticus*, including initial weight (IW), percentage of weight gain (WG), feed conversion ratio (FCR), specific growth rate (SGR), and survival rate (SR), compared to the control treatment (Table 3). Furthermore, the administration of EA at doses of 0.15 and 0.2 g kg^−1^, PP at 15 and 20 g kg^−1^, and all EP treatments significantly increased the final weight (FW) of the tilapia compared to the control and among the treatments. Additionally, the effects of EA at 0.2 g kg^−1^ and EP at 10.01 g kg^−1^ on FW were significantly greater compared to all other treatments (except for the PP20 group compared to EA0.2 and EP 1.5) (Table 3, *p*  < 0.05). No mortality was observed during the experimental period, and a 100% SR (Table 3) was observed in all treatments.

### 3.2. Hematological Parameters

There were no significant differences among the treatments for each of the measured blood parameters (i.e., RBC, HB, and HCT), except for the WBC count in the EA 0.2 g and EP 2 treatments, which were both higher than all other treatments (Table 4, *p*  < 0.05).

### 3.3. Serum Biochemical and Antioxidant Parameters

The changes in serum biochemical parameters by EA, PP, and their combination (EP) in *O. niloticus*, including ALP, ALT, AST, T Prot, Alb, Glob, Alb/Glb ratio (Alb/Glb) and glucose levels were not significantly different among the 10 treatments (Table 5).

**Figure 1:**
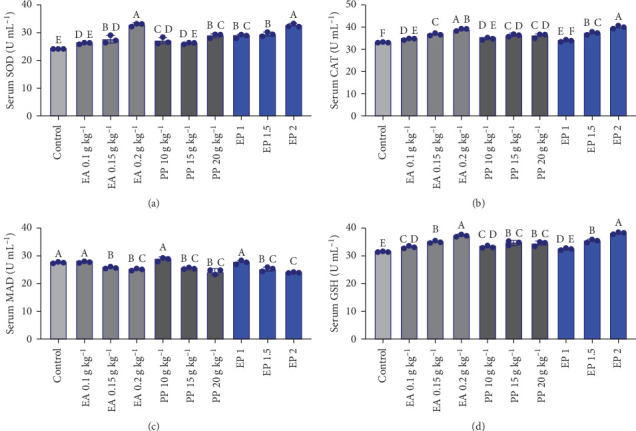
(A–D) Effect of the individual administration of EA and PP, as well as the combination of EA with PP (EP), on the antioxidant parameters of *O. niloticus*. EA: ellagic acid, PP: pomegranate peel, EP: ellagic acid + pomegranate peel. EP 1: 0.05 g + 5 g kg^−1^, EP 1.5: 0.075 g + 7.5 g kg^−1^, and EP 2: 0.1 g + 10 g kg^−1^. Different capital letters signify statistically significant differences. The data are presented as mean values ± SD, and each treatment consists of a pooled sample size of 3 (pooled among tanks) per treatment.

The antioxidant parameters, including SOD, MDA, CAT, and GSH, showed significant responses to the administration of EA, PP, and their combination (EP) in *O. niloticus* (Figure 1A–D). Serum SOD significantly increased in all treatments compared to the control treatment, except for EA 0.1 g and PP 15 g (Figure 1A, *p*  < 0.05), and MDA showed a significantly decreasing trend in all treatments except for EA 0.1 g and PP 10 g kg^−1^ (Figure 1C, *p*  < 0.05). The CAT and GSH enzymes showed significant changes from their values in the control treatment, except that of EP 1 (5.05 g kg^−1^) (Figure 1B,D, *p*  < 0.05).

### 3.4. Immunological Parameters

**Figure 2:**
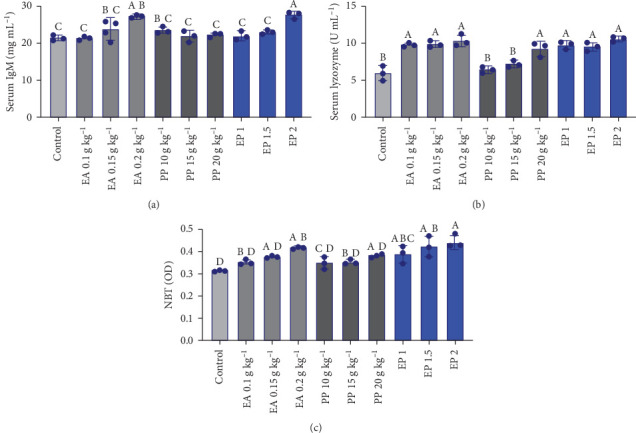
(A–C) Effect of the individual administration of EA and PP, as well as the combination of EA with PP (EP), on the immunological parameters of *O. niloticus*. EA: ellagic acid, PP: pomegranate peel, EP: ellagic acid + pomegranate peel. EP 1: 0.05 g + 5 g kg^−1^, EP 1.5: 0.075 g + 7.5 g kg^−1^, and EP 2: 0.1 g + 10 g kg^−1^. Different capital letters indicate a significant difference. The data are presented as mean values ± SD and a pooled sample size of 3 per treatment.

Overall, the immune activity of *O. niloticus* was shown to be significantly affected by supplementation of EA, PP, and EP at various concentrations of inclusion in feed treatments. Serum IgM did not show any significant differences among most of the dietary supplement treatments compared to the control treatment (Figure 2A, *p* > 0.05), except for the EA 0.2 g kg^−1^ and EP 1 (5.05 g kg^−1^) treatments, which exhibited a significant increase (Figure 2A, *p*  < 0.05). Serum lysozyme levels were significantly higher (*p*  < 0.05) in all supplementary treatments of EA, PP, and EP compared to the control treatment, while both the PP 10 and 15 g kg^−1^ treatments (Figure 2B, *p*  < 0.05). RBA was significantly increased in the samples from EA 0.2 g kg^−1^ and all EP treatments compared to the control (Figure 2C, *p*  < 0.05), while there were no significant differences observed in any of the PP treatments compared to the control treatment (Figure 2C, *p* > 0.05).

### 3.5. Digestive Enzymes

**Figure 3:**
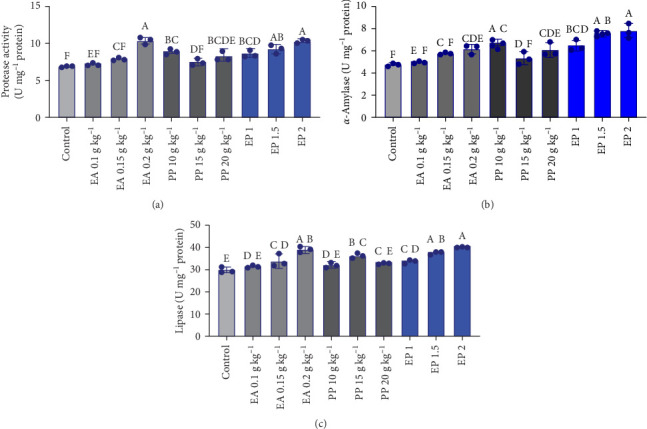
(A–C) Effect of the individual administration of EA and PP, as well as the combination of EA with PP (EP), on the digestive enzymes of *O. niloticus*. EA: ellagic acid, PP: pomegranate peel, EP: ellagic acid + pomegranate peel. EP 1: 0.05 g + 5 g kg^−1^, EP 1.5:0.075 g + 7.5 g kg^−1^, and EP 2: 0.1 g + 10 g kg^−1^. Different capital letters represent statistically significant differences. The data are presented as mean values ± SD and a pooled sample size of 3 per treatment.

Overall, following the administration of EA, PP, and their combination (EP), the digestive enzyme activity (i.e., protease, lipase, and *α*-amylase) in *O. niloticus* showed a significant increase compared to the control treatment. The protease and *α*-amylase activity significantly increased in EA 0.2 g kg^−1^, PP (10 and 20 g kg^−1^), and in all EP treatments, compared to the control treatment (Figure 3A,B; *p*  < 0.05). In the case of lipase, the EA at doses of 0.15 g and 0.2 g kg^−1^, PP at 15 g kg^−1^, and all treatments of EP resulted in a significant increase compared with the control group (Figure 3C, *p*  < 0.05). Overall, a higher level of all three enzymes was observed in EA treatments.

### 3.6. Gene Expression

**Figure 4:**
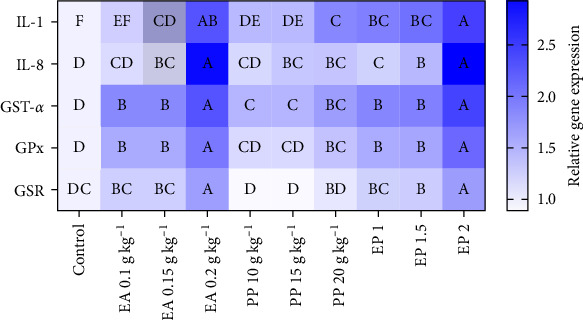
Effect of the individual administration of EA and PP, as well as the combination of EA with PP (EP), on the gene expression of *O. niloticus*. EA: ellagic acid, PP: pomegranate peel, EP: ellagic acid + pomegranate peel. EP 1: 0.05 g + 5 g kg^−1^, EP 1.5: 0.075 g + 7.5 g kg^−1^, and EP 2: 0.1 g + 10 g kg^−1^. Different capital letters refer to statistically significant differences. The data are presented as mean values ± SD, and a pooled sample size of 3 per treatment.

The relative expression levels of immune (IL-1, IL-8, GST-*α*) and antioxidant (GPx and GSR) genes in Nile tilapia showed significant differences following the administration of EA, PP, and EP (Figure 4). The relative expression level of IL-1 showed a significant increase in all treatments except for EA 0.1 g kg^−1^ and IL-8 showed a significant increase in all treatments except for EA 0.1 g and PP 10 g kg^−1^. GST-*α* transcripts were upregulated in all treatments, whereas GPx transcripts were upregulated in all treatments except for PP 10 and 15 g kg^−1^. GSR expression levels were significantly higher in EA 0.2 g, EP 1.5 (7.575 g kg^−1^), and EP 2 (10.01 g kg^−1^) compared to the control treatment (Figure 4, *p*  < 0.05).

## 4. Discussion

In this study, the effect of the pure polyphenol and PP as a natural source of polyphenol from agricultural by-products was investigated on Nile tilapia by measuring a wide range of parameters, including growth metrics, hematological indices, serum biochemical markers, antioxidant activity, immunological responses, digestive enzyme activity, and genes expression to better understand positive and any potential adverse effects. The phenolic compounds, including EA, have been documented to possess antioxidant, anti-inflammatory, and other biological effects that help shield both animals from oxidative stress [51] alongside potential anti-nutrient effects. Consequently, the aquaculture sector increasingly advocates the adoption of phytoextracts as viable alternatives to conventional chemotherapy, promoting a sustainable and health-conscious approach to aquaculture management.

In the present study, the individual and combined administration of EA and PP at various inclusions did not show an adverse effect on growth parameters (FCR, SGR, and WG) in *O. niloticus*. Even administering EA at doses of 0.15 and 0.2 g kg^−1^, PP at 15 and 20 g kg^−1^, and EP for all treatment concentrations resulted in increased FW of the tilapia. Growth performance serves as a primary indicator of the effectiveness of dietary strategies in aquaculture, commonly assessed through parameters like weight increase, growth rate, and condition factor [52]. The positive effect of PP on growth has been reported in broiler chickens in lower doses [24]. Some other studies have also reported a notable increase in growth performance of *Labeo rohita* [33], *O. niloticus* [28], *L. rohita* [53], *Carassius auratus* [32], and *Ctenopharyngodon idella* [54] fed with dietary supplementation of PP extract. In *P. fulvidraco*, the administration of EA led to a notable increase in FW, WG (%), and SGR while not resulting in a notable change in FCR [27]. Similarly, EA at doses of 50, 100, and 200 mg did not significantly affect growth parameters, including FW, FCR, SR, WG, and SGR, in *Oncorhynchus mykiss* [25, 28]. In the current study, the delivery of PP in all doses resulted in similar effects on growth factors in tilapia as EA in most cases, supportive of its safety and effectiveness as a supplement in fish feed. Examining digestive enzyme activities offers valuable insights into how nutrients influence digestive processes and nutrient absorption, both of which are vital for the overall health and growth [16, 55]. Overall, the application of EA, PP, and their combination in this study led to an increase in digestive enzymes. The administration of EA at 0.2 g, PP at 10, 15 (except for protease and *α*-amylase), and 20 g, and EP in all treatments significantly increased the activities of digestive enzymes, including protease and *α*-amylase. Additionally, EA (0.15 g and 0.2 g), PP (15 g), and all EP treatments notably enhanced lipase activity. Although studies on the effects of EA and PP in combination on digestive enzyme activity are limited, the few available studies that have used them separately have produced results that are consistent with the findings of the current study. For example, EA significantly enhanced the activity of digestive enzymes in yellow-feathered broilers, including chymotrypsin, pepsin, and lipase, while amylase showed no significant change [55]. EA also significantly increased the activity of digestive enzymes such as lactase and sucrase in mice [56]. In another study, with medicinal fruits, mandarin peel powder increased amylase enzyme activity in *O. niloticus* [57]. This effect may result from the beneficial effects of polyphenol properties for gut microbiota, triggering the production of enzyme synergistic interactions, improving gut health, and facilitating better nutrient utilization by fish [26, 58]. The slightly higher levels of the digestive enzymes in high EA doses (0.2 g) than observed in the PP treatments may reflect the potential antinutrient (e.g., tannins) in PP, or lower than the estimated content of polyphenols in PP. However, this was not remarkably different among all PP treatments. The EA content in PP is reported to be around (0.39–3.04 mg g^−1^ dry weight, and 1 mg g^−1^) on average [34, 59]. However, the EA content of PP can vary more depending on fruit cultivar and growing conditions. Moreover, there may also be variations in the chemical form of EA in pomegranate [6].

EA, as a polyphenol present in PP, is reported to reduce harmful gut microorganisms by interfering with oxidative phosphorylation, thereby changing the gut environment in favor of beneficial microorganisms [60]. These microorganisms can enhance the availability and absorption of nutrients in the intestinal tract and improve nutrient digestion [26]. The absence of adverse effects from EA and PP on the growth and the digestive system of fish in this study supports their safe use as immuno-stimulants in fish without adverse impacts. These two compounds together (EP) seemed to synergistically improve WG, likely due to the combined effect of EA with other compounds in PP.

In this current study, there were no adverse effects from PP on blood biomarkers of Nile tilapia, with no significant deviation for blood parameters compared to the control treatment. A significant alteration in hematological biomarkers HCT, RBCs, and HB, as well as WBCs (both innate and adaptive immune responses by expressing cell-specific immune-relevant genes), has been recorded in some medicinal plant trials in fish [61, 62]. The rise in WBCs seen with EA and EP is attributed to the boost in the immune system and decreased oxidative stress, without any adverse hematological parameters.

The results of the current study showed no significant changes in serum biochemical parameters by EA, PP, and their combination (EP) in *O. niloticus*, including ALP, ALT, AST, T Prot, Alb, Glob, Alb/Glb, and glucose levels. Blood biochemistry parameters, such as AST, ALT, and ALP, are indicator enzymes of liver function that respond to nutritional and environmental conditions [63]. In line with our results, no significant effect of PP on biochemical parameters such as ALT and AST was reported in *O. niloticus* [28]. However, administering olive leaf extract to common carp orally at doses of 0.1% and 0.2% [64] as well as Indian quince in [65] resulted in elevated liver enzymes, including AST, ALT, and ALP, leading to hepatotoxicity in the fish. The lack of significant changes in biochemical parameters when compared to the control group suggests that the health status of the fish remains stable after administration of the compounds studied and therefore, the treatments did not induce any adverse effects. The antioxidant defense system plays a crucial role in fish health by reducing the harmful effects of free radicals and oxidative stress. Oxidative stress from environmental challenges and pathogens can compromise the immune system [66]. Our results showed that significant serum antioxidants, namely SOD, CAT, and GSH levels, were elevated in all supplementary treatments, especially those fish receiving a combination of EA and PP. Additionally, serum MDA levels in EA (0.15 and 0.2 g), PP (15 and 20 g), and EA (1.5 and 2) groups were significantly lower than those in the control group. The expression of key antioxidant-related genes, including GST-*α* (in all treatments), GPx (except PP 10 and 15 g treatments), and GSR (in EA 0.2 g, EP 1.5, and EP 2 treatments), was also significantly upregulated, reflecting improved antioxidant capacity.

Consistent with the results of our study, it is reported that PP led to a significant increase in antioxidant enzymes, such as CAT, GSH, and SOD, and a significant decrease in MDA in *O. niloticus* [28]. Similarly, changes in CAT, SOD, and GSH MDA levels were also observed in the liver of *O. mykiss* exposed to a similar compound [31] and were associated with a protective effect of these antioxidants in multiple tissues, including the liver, kidney, and spleen of *O. mykiss* [25]. The increase in CAT, SOD, and total antioxidant capacity was reported using EA in *P. fulvidraco* [27], and the impact of PP polyphenols on antioxidant enzymes and gene expression in *T. ovatus* [67] has also been reported. However, the lack of significant change in the activity of CAT, SOD, and MDA enzyme levels was reported using EA in *C. carpio* liver [68]. Additionally, this compound significantly boosted the expression of CAT, SOD, and glutathione peroxidase genes when compared to the control treatment [67]. It has been found that fermented PP polyphenols significantly influenced antioxidant enzyme activities (SOD, CAT, GSH, and MDA) and enhanced the expression of antioxidant genes (SOD and CAT) in *Litopenaeus vannamei* [69]. This antioxidant activity allows immune cells to operate more effectively by scavenging free radicals and preventing cellular damage [70]. Moreover, an ameliorative effect on hepatic damage and reduced glutathione depletion in zebrafish is attributed to EA administration [29]. The increase in the activity of antioxidant enzymes and their gene's expressions in Nile tilapia treated with EA and PP is attributed to the abundant bioactive compounds in PP, including phenolics and flavonoids, which boost antioxidant activity [71]. The immune system plays an essential role in resistance to disease, which is critical to overall fish health and can be modulated by functional feed additives. In this study, the WBC count significantly increased in the EA 0.2 g and EP 2 treatments. In previous studies, the methanolic extract of PP increased WBCs in *O. niloticus* [72] and *Cyprinus carpio* [73]. The increase in WBCs observed with EA and EP is likely due to their immunomodulatory role while reducing oxidative stress. Furthermore, lysozyme activity, IgM, and NBT levels were significantly increased in the EA 0.2 g and EP 2 treatments, indicating enhanced innate immune function. This suggests that higher doses of EA alone or in combination with PP (EP) may enhance innate immune responses as observed for polyphenols, for example, [74]. An increase in immune parameters such as lysozyme, IgM, RBA, and phagocyte activity was reported in *O. mykiss* using EA [25]. These outcomes could be linked to the bioactive constituents found in EA and PP, which are known for their potential to stimulate the immune system. EA has antioxidant properties, and PP is rich in antioxidant compounds, which reduce oxidative stress, thereby improving the health and functionality of immune cells. Furthermore, EA and PP exhibit immunomodulatory effects by stimulating the production of immune-related molecules, leading to increased levels of lysozyme and IgM. An increase in RBA and lysozyme activity enhances the antimicrobial functions of macrophages and immune cells [75].

In line with the factors described above (i.e., lysozyme, RBA, and IgM), the expression of immune-related genes, including IL-1 and IL-8, was also significantly upregulated in response to the supplements, further supporting the immuno-stimulatory effects of EA and EP. Elevated IL-1 levels were observed in all treatments except EA 0.1 g, and elevated IL-8 levels in all treatments except EA 0.1 mg and PP 10 g indicated immune system modulation by EA and PP. The significant rise in IL-1 and IL-8 expression suggests activation of pattern recognition receptors and immune components, indicating a stimulated early inflammatory response [33]. This enhancement is vital for the innate immune response, providing better defense against pathogens. Additionally, the polyphenolic compounds in these extracts can upregulate the expression of immune-related genes such as IL-1 and IL-8, which are crucial for initiating inflammatory responses and regulating immune signaling pathways [76].

In *Labeo rohita*, dietary supplementation with PP extract improved several immune parameters, including lysozyme, RBA, and total immunoglobulin [33]. The current study also found that PP significantly enhanced immune parameters, including lysozyme, IgM, and RBA, in *O. niloticus*. In a separate study investigating the effect of PP polyphenols at different doses on immunity and the expression of immunity-related genes in *Trachinotus ovatus*, dietary supply of PP at 270 mg kg^−1^ significantly enhanced serum lysozyme, C3, and C4 levels, as well as the expression of the IL-10 gene across all levels, while having no effect on serum IgM [67]. The use of PP has also resulted in a notable rise in serum lysozyme, antiprotease activity, and nitric oxide levels in catfish *Clarias gariepinus* when compared to a control treatment [77]. The synergy between EA and these compounds further amplifies the antioxidant response, leading to greater protection against oxidative stress. These synergistic mechanisms require further investigations to contribute to more insightful conclusions and to lead to optimized dosages for application in aquaculture.

## 5. Conclusion

This study highlights the potential of EA, PP powder, and their combination for improving growth performance, immune response, antioxidant activity, and gene expression in Nile tilapia (*O. niloticus*) over a 60-day feeding trial. The supplementation at higher doses significantly enhanced immune parameters, such as WBCs and lysozyme levels. Furthermore, the antioxidant enzyme activities, including SOD, CAT, and glutathione, were elevated across treatments. Notably, the expression of immune-antioxidant genes was significantly increased in all supplementary treatments, reflecting the potential of these natural immune stimulants to improve the overall health and growth of Nile tilapia without adversely affecting feed conversion, growth, or SRs or the digestive system. EA and PP enhance intestinal digestive enzymes (protease, *α*-amylase, and lipase), which promote better nutrient digestion and absorption in aquatic diets. These findings suggest the use of PP and even that incorporating EA and PP synergistically into aquaculture diets could serve as a viable strategy for enhancing fish growth and immunity, contributing to sustainable aquaculture practices. Also, it is recommended that future studies evaluate higher doses of supplements while assessing their toxicity and potential adverse effects.

## Figures and Tables

**Table 1:** Formulation and proximate composition of 10 dietary treatments for *O. niloticus*.

Ingredients	Control (basal g kg^−1^)	EA 0.1 g kg^−1^	EA 0.15 g kg^−1^	EA 0.2 g kg^−1^	PP 10 g kg^−1^	PP 15 g kg^−1^	PP 20 g kg^−1^	EP 1 (5.05 g kg^−1^)	EP 1.5 (7.575 g kg^−1^)	EP 2 (10.01 g kg^−1^)
Fish meal	200	200	200	200	200	200	200	200	200	200
Soybean meal	290	290	290	290	290	290	290	290	290	290
Corn meal	240	240	240	240	240	240	240	240	240	240
Wheat flour	130	130	130	130	130	130	130	130	130	130
Rice bran	106.5	106.4	106.35	106.3	96.5	91.5	86.5	101.45	98.925	96.4
Fish oil	6.75	6.75	6.75	6.75	6.75	6.75	6.75	6.75	6.75	6.75
Soybean oil	6.75	6.75	6.75	6.75	6.75	6.75	6.75	6.75	6.75	6.75
Ellagic acid	0	0.1	0.15	0.2	0	0	0	0	0	0
Pomegranate	0	0	0	0	10	15	20	0	0	0
Ellagic acid + pomegranate peel powder	0	0	0	0	0	0	0	5.05	7.575	10.1
Vitamin and mineral premix^a^	15	15	15	15	15	15	15	15	15	15
Gelatin	5	5	5	5	5	5	5	5	5	5
Total	1000	1000	1000	1000	1000	1000	1000	1000	1000	1000

Crude protein (%)	33.23	32.94	32.91	32.78	33.14	33.05	33.01	33.09	32.93	32.84
Fat (%)	9.71	9.67	9.65	9.62	9.44	9.44	9.40	9.40	9.41	9.39
Crude fiber (%)	5.9	5.9	6.1	6.4	6.7	6.9	7.1	6.8	7.1	7.3
Ash (%)	9.1	9	8.9	8.9	9.3	9.5	9.6	9.3	9.6	9.8
Moisture (%)	9.1	9.1	9.2	9.2	9.4	9.5	9.7	9.4	9.6	9.8
NFE^b^ (%)	32.96	33.39	33.24	33.10	32.02	31.61	31.19	32.01	31.36	30.87
GE^c^ (MJ kg^−1^)	17.34	17.33	17.29	17.22	17.05	16.96	16.86	17.02	16.88	16.76

*Note:* EP: ellagic acid + pomegranate peel.

Abbreviations: EA, ellagic acid; PP, pomegranate peel.

^a^The vitamin and mineral premix, sourced from Lajvar Aquatic Feed Mill, Iran, fulfills the nutritional needs of *O. niloticus*. The composition of the vitamin and mineral premix includes the following: 1000 IU of vitamin A, 250 IU of vitamin D3, 5 IU of vitamin E, 2000 mg of vitamin B1, 800 mg of vitamin B2, 2000 mg of vitamin B6, 1 mg of vitamin B12, 10,000 mg of vitamin C, 300 mg of pantothenic acid, 5000 mg of nicotinic acid, 800 mg of manganese, 10 mg of selenium, 15,000 mg of lysine, and 3000 mg of methionine.

^b^Nitrogen-free extract = 100 − (CP + fat + crude fiber + ash + moisture).

^c^Gross energy = (CP × 23.6) + (fat × 39.5) + (NFE × 17.2).

**Table 2:** The specifications of primers utilized for qPCR analysis of mRNA expression in the liver tissue of *O. niloticus* in this study.

Genes	Sequence (5′ – 3′)	Ta	Accession no.	Product size	Efficiency (%)	Ref.
*β*-Actin	AGCAAGCAGGAGTACGATGAG TGTGTGGTGTGTGGTTGTTTTG	62	KJ126772.1	203	98.9	[48]
Interleukin-1	GTCTGTCAAGGATAAGCGCTG ACTCTGGAGCTGGATGTTGA	59	XM_019365844	200	95	[49]
Interleukin-8	CTGTGAAGGCATGGGTGTG GATCACTTTCTTCACCCAGGG	59	NM_001279704	196	97.5	[49]
Glutathione S-transferase-*α*	ACTGCACACTCATGGGAACA TTAAAAGCCAGCGGATTGAC	60	NM_001279635	190	96.8	[49]
Glutathione peroxidase	GGTGGATGTGAATGGAAAGG CTTGTAAGGTTCCCCGTCAG	60	NM_001279711	190	95	[49]
Glutathione reductase	CTGCACCAAAGAACTGCAAA CCAGAGAAGGCAGTCCACTC	60	XM_005467348	172	96.6	[49]

**Table 3:** Growth parameters for *O. niloticus* grown for 60 days on 10 dietary treatments incorporating various concentrations of either EA, PP, or a combination of both, as well as a control treatment without inclusion.

Treatments	Control (basal g kg^−1^)	EA 0.1 g kg^−1^	EA 0.15 g kg^−1^	EA 0.2 g kg^−1^	PP 10 g kg^−1^	PP 15 g kg^−1^	PP 20 g kg^−1^	EP 1 (5.05 g kg^−1^)	EP 1.5 (7.575 g kg^−1^)	EP 2 (10.01 g kg^−1^)
IW (g)	10.33 ± 0.57	10.00 ± 1.00	10.66 ± 0.57	10.00 ± 1.00	10.33 ± 1.52	10.66 ± 0.57	10.66 ± 1.52	9.66 ± 0.57	10.33 ± 0.57	11.00 ± 1.00
FW(g)	48.83 ± 0.76^D^	50.50 ± 0.50^CD^	51.16 ± 1.04^C^	53.66 ± 0.76^AB^	50.33 ± 0.28^CD^	51.33 ± 0.57^C^	51.83 ± 0.28^BC^	51.33 ± 0.28^C^	52.00 ± 1.00^BC^	54.50 ± 0.50^A^
WG (%)	373.78 ± 32.50	408.21 ± 48.25	380.30 ± 17.20	440.63 ± 60.30	393.79 ± 68.05	382.12 ± 24.56	393.22 ± 76.36	432.22 ± 29.82	404.54 ± 35.77	398.05 ± 42.94
WG (g)	38.50 ± 1.32^B^	40.50 ± 0.86^AB^	40.50 ± 0.50^AB^	43.66 ± 1.60^A^	40.00 ± 1.32^B^	40.66 ± 0.57^AB^	41.16 ± 1.75^AB^	41.66 ± 0.28^AB^	41.66 ± 1.52^AB^	43.50 ± 0.86^A^
FCR (g g^−1^)	0.95 ± 0.06	0.89 ± 0.08	0.93 ± 0.03	0.83 ± 0.09	0.92 ± 0.13	0.93 ± 0.04	0.92 ± 0.13	0.84 ± 0.04	0.89 ± 0.06	0.90 ± 0.07
SGR (% day ^−1^)	2.58 ± 0.11	2.70 ± 0.15	2.61 ± 0.05	2.80 ± 0.18	2.65 ± 0.23	2.62 ± 0.08	2.64 ± 0.25	2.78 ± 0.09	2.69 ± 0.12	2.67 ± 0.14
SR (%)	100 ± 0.00	100 ± 0.00	100 ± 0.00	100 ± 0.00	100 ± 0.00	100 ± 0.00	100 ± 0.00	100 ± 0.00	100 ± 0.00	100 ± 0.00

*Note:* EP: EA + PP. Differences between means within the same row are indicated by different superscript letters (*p*  < 0.05). The data are presented as mean values ± SD and consist of a sample size of *n* = 45 per treatment. IW: initial weight of the fish at the beginning of the experiment, FW: weight of the fish at the end of the trial, WG: weight gained during the trial.

Abreviations: EA, ellagic acid; FCR, feed conversion rate; PP, pomegranate peel; SGR, specific growth rate; SR, survival rate.

**Table 4:** Effect of dietary treatments containing either EA, PP, or a combination of both, as well as a control treatment without inclusion, on the hematological indices of *O. niloticus*.

Treatments	Control (basal g kg^−1^)	EA 0.1 g kg^−1^	EA 0.15 g kg^−1^	EA 0.2 g kg^−1^	PP 10 g kg^−1^	PP 15 g kg^−1^	PP 20 g kg^−1^	EP 1 (5.05 g kg^−1^)	EP 1.5 (7.575 g kg^−1^)	EP 2 (10.01 g kg^−1^)
WBCs (10^3^ µL^−1^)	5.33 ± 0.05^BC^	6.06 ± 0.20^BC^	5.80 ± 0.36^BC^	8.36 ± 1.23^A^	5.23 ± 0.15^BC^	6.06 ± 0.20^BC^	5.76 ± 0.25^BC^	6.13 ± 0.20^BC^	6.63 ± 0.75^B^	8.30 ± 0.20^A^
RBCs (10^6^ µL^−1^)	1.33 ± 0.11	1.33 ± 0.15	1.43 ± 0.05	1.56 ± 0.05	1.50 ± 0.17	1.60 ± 0.26	1.56 ± 0.15	1.40 ± 0.10	1.40 ± 0.10	1.40 ± 0.20
HB (g dL^−1^)	9.5 ± 0.36	9.6 ± 0.26	9.73 ± 0.28	9.70 ± 0.20	9.46 ± 0.11	9.66 ± 0.40	9.40 ± 0.10	9.76 ± 0.30	9.33 ± 0.15	9.36 ± 0.15
HCT (%)	29.00 ± 2.64	30.66 ± 3.21	30.66 ± 2.51	31.33 ± 3.51	30.00 ± 4.35	28.66 ± 4.50	30.00 ± 2.64	30.00 ± 1.00	30.33 ± 1.52	31.66 ± 1.52
Neutrophils (%)	15.66 ± 0.57	16.00 ± 1.00	16.66 ± 0.57	16.66 ± 0.57	16.66 ± 0.57	16.66 ± 0.57	16.66 ± 0.57	16.66 ± 0.57	16.66 ± 1.52	17.33 ± 1.15
Lymphocytes (%)	77.33 ± 0.57	77.00 ± 1.00	76.66 ± 1.15	76.66 ± 1.15	76.66 ± 0.57	76.33 ± 0.57	76.00 ± 1.00	76.33 ± 0.57	77.00 ± 1.00	77.33 ± 2.00
Monocytes (%)	6.00 ± 1.00	6.00 ± 1.00	4.33 ± 2.08	4.66 ± 1.15	5.66 ± 0.57	5.33 ± 0.57	5.33 ± 0.57	5.00 ± 1.00	4.33 ± 1.15	5.00 ± 0.00
Eosinophils (%)	1.00 ± 1.00	1.00 ± 1.00	2.33 ± 0.57	2.00 ± 1.00	1.00 ± 1.00	1.66 ± 0.57	2.00 ± 0.00	2.00 ± 0.00	2.00 ± 0.00	1.33 ± 0.57

*Note:* EP: EA + PP. Significant differences between means (from the pooled sample size of 3 per treatment) within the same parameter (row) are indicated by different superscript letters (*p*  < 0.05).

Abbreviations: EA, ellagic acid; HB, hemoglobin; HCT, hematocrit; PP, pomegranate peel; RBCs, red blood cells count; WBCs, white blood cells count.

**Table 5:** Effect of dietary treatments containing either EA, PP, or a combination of both, as well as a control treatment without inclusion, on the serum biochemical indices of *O. niloticus*.

Treatments	Control (basal g kg^−1^)	EA 0.1 g kg^−1^	EA 0.15 g kg^−1^	EA 0.2 g kg^−1^	PP 10 g kg^−1^	PP 15 g kg^−1^	PP 20 g kg^−1^	EP 1 (5.05 g kg^−1^)	EP 1.5 (7.575 g kg^−1^)	EP 2 (10.01 g kg^−1^)
ALP (U L^−1^)	31.40 ± 1.36	30.21 ± 2.80	30.58 ± 2.50	31.07 ± 1.92	29.80 ± 1.81	30.50 ± 2.96	28.22 ± 2.41	28.83 ± 0.69	29.79 ± 0.74	29.95 ± 0.21
ALT (U L^−1^)	6.73 ± 0.72	6.85 ± 0.10	6.80 ± 0.30	6.87 ± 0.20	6.81 ± 0.21	6.99 ± 0.41	6.71 ± 0.14	6.87 ± 0.23	6.95 ± 0.06	6.97 ± 0.33
AST (U L^−1^)	173.17 ± 3.99	168.23 ± 13.0	172.92 ± 4.32	172.65 ± 4.87	171.58 ± 6.20	171.08 ± 5.43	171.41 ± 4.79	172.35 ± 2.10	170.57 ± 2.66	170.04 ± 5.08
T Prot (g dL^−1^)	3.62 ± 0.06	3.66 ± 0.37	3.78 ± 0.07	3.70 ± 0.26	3.68 ± 0.14	3.73 ± 0.62	3.71 ± 0.32	3.73 ± 0.23	3.81 ± 0.23	3.86 ± 0.33
Alb (g dL^−1^)	1.61 ± 0.03	1.45 ± 0.44	1.63 ± 0.03	1.63 ± 0.02	1.61 ± 0.05	1.67 ± 0.08	1.67 ± 0.05	1.61 ± 0.07	1.63 ± 0.03	1.66 ± 0.10
Glob (g dL^−1^)	2.00 ± 0.03	2.21 ± 0.81	2.14 ± 0.07	2.07 ± 0.24	2.06 ± 0.20	2.06 ± 0.67	2.04 ± 0.34	2.12 ± 0.19	2.17 ± 0.27	2.19 ± 0.32
Alb/Glb (g dL^−1^)	0.80 ± 0.01	0.76 ± 0.45	0.76 ± 0.03	0.79 ± 0.08	0.78 ± 0.10	0.88 ± 0.33	0.84 ± 0.17	0.76 ± 0.06	0.76 ± 0.11	0.77 ± 0.13
Glucose (mg dL^−1^)	52.44 ± 0.83	51.90 ± 1.55	52.32 ± 1.34	52.92 ± 0.70	52.84 ± 1.50	52.38 ± 1.40	53.38 ± 1.29	52.64 ± 2.27	52.90 ± 2.01	52.94 ± 0.28

*Note:* EP: EA + PP.

Abbreviations: Alb, albumin; ALP, alkaline phosphatase; ALT, alanine transaminase; AST, aspartate transferase; EA, ellagic acid; Glob, globulin; PP, pomegranate peel; T Prot, total protein.

## Data Availability

Data will be made available upon request.
